# Supplementary figures and images for: A large-scale brain network mechanism for increased seizure propensity in Alzheimer’s disease
Source: PLoS Comput Biol. 2021 Aug 11;17(8):e1009252. doi: 10.1371/journal.pcbi.1009252 (PMC8382184; doi:10.1371/journal.pcbi.1009252)

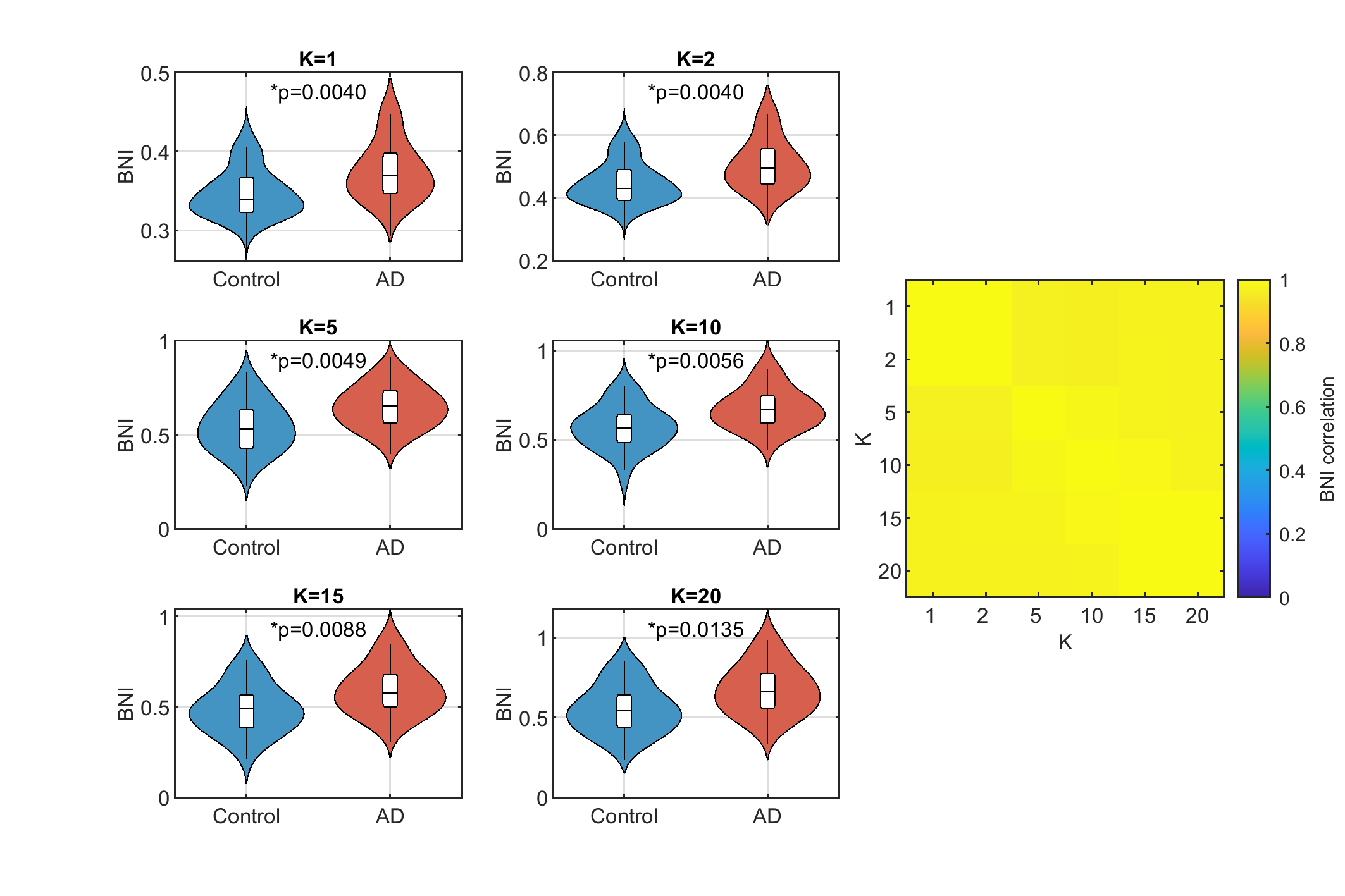

Supplement: S1 Fig — The plots on the left and centre recreate Fig 2 for a range of values of global coupling constant K. The correlation matrix on the right shows Spearman’s correlation of BNI scores across participants as different values of K are used. All correlations were ≥ 0.9665. (TIF) [file pcbi.1009252.s001.tif]

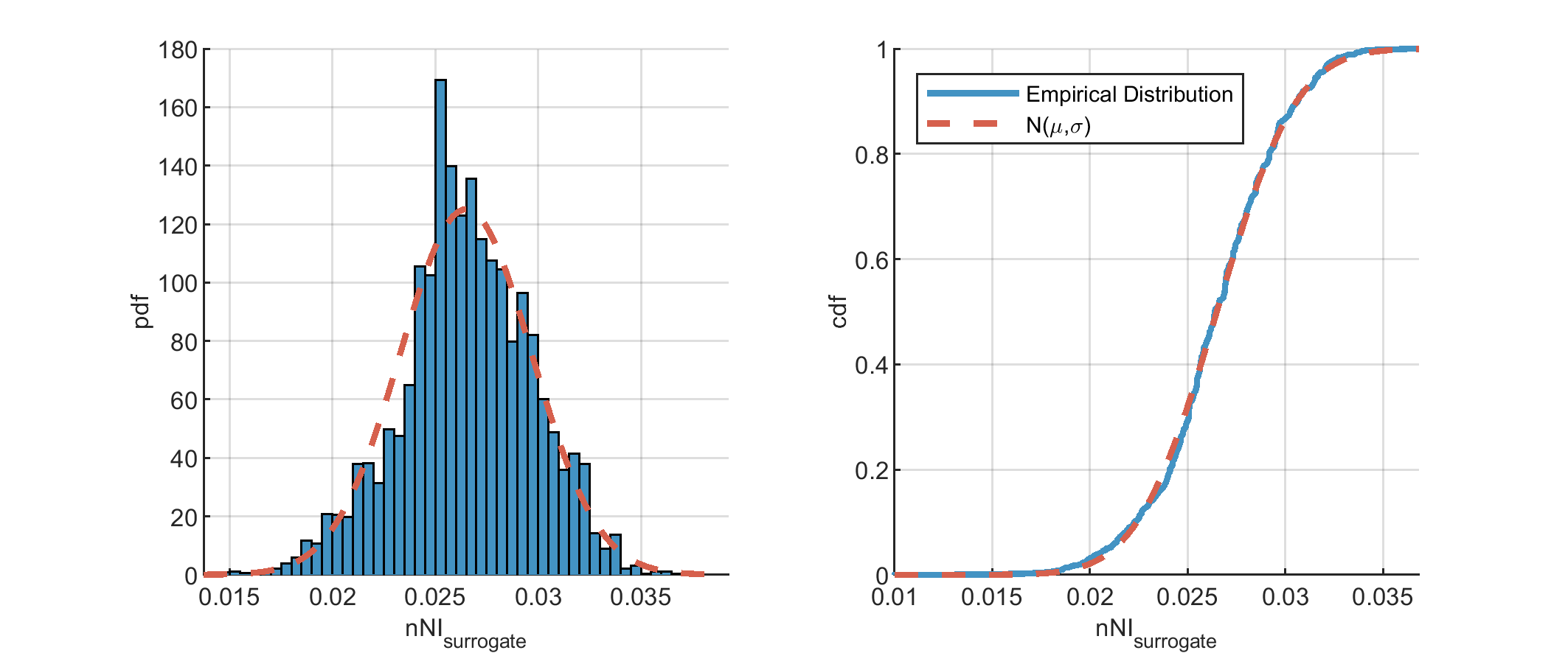

Supplement: S2 Fig — Probability density function (pdf; left) and cumulative distribution functions (cdf; right) for the empirical data and best fit normal distribution. A Kolmogrov-Smirnov test showed no significant differences between the empirical and normal distributions (p = 0 using Matlab’s kstest function). (TIF) [file pcbi.1009252.s002.tif]
