# Supplementary material for: A large-scale brain network mechanism for increased seizure propensity in Alzheimer’s disease
Source: PLoS Comput Biol. 2021 Aug 11;17(8):e1009252. doi: 10.1371/journal.pcbi.1009252 (PMC8382184; doi:10.1371/journal.pcbi.1009252)
Supplement: S1 Table — For each ROI, we give a full name, and the abbreviation used in Fig 3. (PDF) [file pcbi.1009252.s003.pdf]

ROI	Abbreviation	ROI	Abbreviation
Frontal lobes			
Superior frontal gyrus	sup front	Middle frontal gyrus	Mid front
Inferior frontal gyrus	inf front	Orbital gyrus	orbital
Precentral gyrus	precent	Paracentral gyrus	paracent
Temporal lobes			
Superior temporal gyrus	sup temp	Middle temporal gyrus	mid temp
Inferior temporal gyrus	inf temp	Fusiform gyrus	fusiform
Parahippocampal gyrus	parahipp	Posterior superior temporal sulcus	post sts
Parietal lobe			
Superior parietal lobule	sup parietal	Inferior parietal lobule	inf parietal
Precuneus	precuneus	Postcentral gyrus	postcent
Occipital lobe			
Medioventral occipital cortex	medvent occip	Lateral occipital cortex	lat occip
Others			
Insular cortex	insular	Cingulate cortex	cingulate
