# Supplementary material for: A large-scale brain network mechanism for increased seizure propensity in Alzheimer’s disease
Source: PLoS Comput Biol. 2021 Aug 11;17(8):e1009252. doi: 10.1371/journal.pcbi.1009252 (PMC8382184; doi:10.1371/journal.pcbi.1009252)
Supplement: Attachment — Submitted filename: 2021 PLOSCB AD seizurereview.pdf [file pcbi.1009252.s005.pdf]


The authors have successfully addressed most of my comments.

Only some minor comments beneath:

Question 1:

- Old question: “Additionally, the authors seem to be describing a model where excitability and seizure probability increase progressively across disease progression (moderate dementia subjects would have more probability of seizures than MCI subjects), but, is that really the case?”
- Answer from authors: “Evidence in the literature suggests that this is the case. For an example, we refer the reviewer to Fig 1B of Beagle et al. (2017), which shows a plot of the seizure probability against years from disease onset, demonstrating a steady increase over disease progression. We have referred to this in the discussion: “It would be particularly interesting to track the progression of modelled seizure probability as brain networks evolve during disease progression. Indeed, the probability of seizures has been observed to increase steadily from disease onset [70].” (lines 333-335) “
- New comment: From my perspective the reference to Beagle 2017 does not address the question successfully. In the Beagle 2017 the paper, the figures of cumulative probability ex Fig. 1 increase with years from disease of onset, as expected since cumulative probability can only increase over time. However, seizure onset is not more likely at advance disease stages than early disease stages, and shown in their Fig. 3.

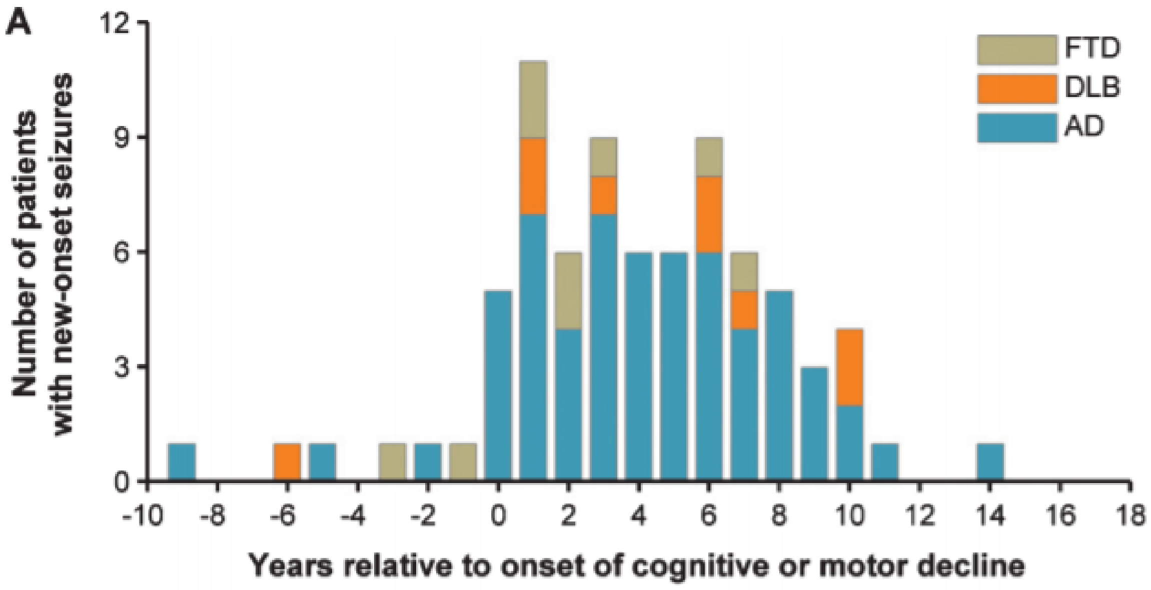

Question 2:

- Old question: Methods for PLV calculation. Line 129 states “ PLV values with zero phase-lag were rejected. Edges with a stronger indirect path were also rejected”. The

phase lag will rarely be exactly zero so I imagine there are some thresholds to assess this. Could you share more details on this?

- Author answer: The threshold for phase lags corresponded to the phase difference corresponding to a single sample at the minimum bandpass frequency (in this case 4 Hz as the theta band ranges from 4-8 Hz). “Zero-phase lag here corresponds to mean phase differences less than the phase difference equal to one sample at 4 Hz.” (lines 134-135)
- New comment. That seems like a not easy to understand sentence. I do personally not understand what this means. Should we deduce what the threshold in phase from the sampling frequency (1kHz?) and a 4Hz sinusoid? Could the authors maybe translate that into a threshold in rads for ex? Or provide a reference that states this more clearly?
-
