# Supplementary material for: A large-scale brain network mechanism for increased seizure propensity in Alzheimer’s disease
Source: PLoS Comput Biol. 2021 Aug 11;17(8):e1009252. doi: 10.1371/journal.pcbi.1009252 (PMC8382184; doi:10.1371/journal.pcbi.1009252)
Supplement: Attachment — Submitted filename: LargeScaleBrainNetMechanism_Response.docx [file pcbi.1009252.s004.docx]

**Reviewer #1**

*The authors perform a relatively simple and straightforward analysis using (1) functional connectivity from resting state EEG data from AD and controls previously published and (2) an existing model for seizure prediction and ictogeneity previously used in epilepsy studies. The manuscript is well written and the work is transparently presented. While there are clear limitations to this study (for ex seizures or epileptiform activity or clinical follow-up not being assessed in these subjects) I believe this paper does add something new to the existing literature of AD. Although there are various papers evaluating hyperexcitability, seizures and epileptiform activity, here the authors bring a different perspective by evaluating the impact of long range connections to seizures.*

*The value of this paper is introducing a mechanism from the epilepsy field to the AD field, involving functional connectivity and its organization. This concept becomes clearer in the results and discussion, but it not very clear in the abstract. At least I felt that the abstract states that the authors are proposing a mechanism but what is this mechanism is unclear. Maybe this could be stated more clearly in the abstract, dedicating 1-2 sentences since is this where the meat of the message is.*

We have updated the abstract as suggested. Relevant additions to the abstract are highlighted in red below. A full list of minor changes to the abstract (due to word count) are given in the tracked changes version of the manuscript:

“*People with Alzheimer's disease (AD) are 6-10 times more likely to develop seizures than the healthy aging population. Leading hypotheses largely consider hyperexcitability of local cortical tissue as primarily responsible for increased seizure prevalence in AD. However, in the general population of people with epilepsy, large-scale brain network organization additionally plays a role in determining seizure likelihood and phenotype. Here, we propose that alterations to large-scale brain network organization seen in AD may contribute to increased seizure likelihood. To test this hypothesis, we combine computational modelling with electrophysiological data using an approach that has proved informative in clinical epilepsy cohorts without AD. EEG was recorded from 21 people with probable AD and 26 healthy controls…*”

*This work is a preliminary test of a hypothesis/mechanisms, and the authors highlight in the discussion and limitations that their dataset is limited so that future studies with a more suited dataset should assess reproducibility and clinical relevance. If that is the goal, it would be important that this work produces a package (meaning methodological description or code) that can be more readily picked up by a future researcher: so that a future researcher can directly apply the same methods than the authors in this manuscript and test their hypothesis. For example the following would be useful:*

We are absolutely in support of this and the code is freely available for academic use, which is now reflected in our code availability statement. This will be managed by a platform that is under development at University of Exeter, but in the mean time it can be requested from the authors. We have included a code availability statement in the manuscript:

*“****Code availability.*** *The model described in this publication is available freely for academic use from the University of Exeter. The underlying source codes are copyright owned by the University of Exeter and can be supplied under licence upon request.”* (lines 205-208)

*- Sharing more details so that the analyses can be reproduced by other researchers (particularly on how to compute the NI and BNI values for each network). The main formulas of the theta model and BNI are presented but some details seem to be missing. How was the simulation performed, what values were used for all the parameters of the model*

To present the parameters more clearly, we have added a new Table 2 which specifies all model parameters, their meaning, and their standard values used in the simulations. We have additionally added the following line to clarify how simulations were performed:

*“Simulations used a stochastic Euler method with time step* $\delta t={10}^{-2}$ *(arbitrary units) and total integration time* $T=4\times{10}^{6}$*.”* (line 163-164)

*- How was the analysis implemented? Custom scripts, toolbox, reference scripts from other researchers, etc?*

The only external toolboxes used were Fieldtrip (for pre-processing and source reconstruction) and MVPA-Lite (for MVPA analysis), which were both cited in the appropriate places (e.g. line 104, lines 106-112, line 225). Otherwise, all scripts (e.g. PLV, BNI simulations, analysis) were custom scripts implemented in Matlab. These custom scripts are now available as per the code availability statement.

*Line 214: the text states: “At baseline excitability (black arrow in Fig 2B), no participant exhibits seizures. This is in line with the observation that no patients studied here exhibit seizures”.(and similar sentence in line 286) Why is that? Does baseline excitability mean Io=lambda1? How was lambda1 selected? Why would lambda1 represent the normal stage of AD subjects and why would the fact that the patients do not have seizures be related to lambda1? How would this look like for an epilepsy patient?*

The reviewer is correct that here “baseline excitability” means $I_{0}=\lambda_{1}$. We wish to quantify the propensity of the networks under study to generate seizures as cortical excitability increases. To do this, we must study changes in the dynamics as I0 is varied. Therefore we have to choose upper and lower bounds for I0 (which we term lambda1 and lambda2). A natural choice for lambda1 is a value at which none of the networks display seizures in the model, as this allows to study the full range of changes in dynamics over increases in I0 (see Fig2B), and is in line with none of the subjects having seizures. Hence $\lambda_{1}$ does not “represent the normal stage of AD subjects”, it represents a non-seizure state for all subjects. This point has been clarified in the main text, which now reads:

”*We first tested whether brain networks in people with AD had a higher propensity to generate seizures than controls by quantifying BNI. BNI was calculated as the area under the curve of percentage of time spent in seizure as cortical excitability (*$I_{0}$*) is increased. The lower bound for* $I_{0}$*, which we term* $\lambda_{1}$*, was chosen to be a value at which no participant exhibits seizures in the simulation. This reflects the fact that at the time of EEG acquisition no participants exhibited seizures. Therefore baseline excitability in the model, i.e.* $I_{0}=\lambda_{1}$ *(black arrow in Fig 2B), represents a non-seizure state for all participants.”* (lines 242-249)

In terms of how this would look for an epilepsy patient, it has been shown that people with epilepsy have higher BNI than controls for wide ranges of the global coupling parameter at fixed excitability [Lopes et al. (2021)]. Since increasing excitability has been shown to have similar effect as increasing the global coupling parameter [Lopes et al. (2017)] we would expect that an epilepsy patient would have lower $\lambda_{1}$ (no seizures in simulations relative to our AD participants. In other approaches, we have previously chosen values of parameters such that the simulation is in the seizure state 50% of the time, and used this as a benchmark to assess hypothetical interventions [Goodfellow et al. (2016)].

*Interpretation of NI and nNI values: What range of values can NI have and what do they mean?*

We have clarified this point in lines 192-201:

“*NI can be interpreted as follows. If node* $i$ *has no influence on seizure generation, then there will be no change in BNI following removal of the node and hence* $BNI_{post}^{\left( i \right)}=BNI_{pre}$ *and* $NI^{(i)}=0$*. Conversely if node* $i$ *is entirely responsible for seizure generation in the network, then seizures are suppressed completely following the removal of the node, and hence* $BNI_{post}^{\left( i \right)}=0$ *and* $NI^{(i)}=1$*. In most real cases, removal of an ROI will reduce seizure propensity but not completely suppress seizures, and hence* $0<BNI_{post}^{\left( i \right)}<BNI_{pre}$ *and* $0<NI^{(i)}<1$*, where larger values indicate seizures are more suppressed following removal of the node. A negative value of* $NI^{(i)}$ *suggests that this node suppresses seizures, and hence removal of the node increases seizure propensity (i.e.* $BNI_{post}^{\left( i \right)}>BNI_{pre}$*)*.”

*Within AD, Increased nNI in regions typically associated to AD pathology, interpreted as these being the regions that drive seizures. However there are decreased NI values AD vs control. Does this mean that AD nodes are less prone to drive seizures than controls? How does this fit together with the previous point*

Lower mean NI values suggest that individual nodes are less likely to drive seizures *in isolation*. More explicitly stated, removing an individual node from the network will (on average) have a lesser effect on reducing seizures in people with AD than controls. This suggests that seizures are less likely to be driven by *individual nodes* in AD, and potentially are more likely to be driven by groups of nodes (i.e. the seizure generating mechanisms are more distributed). We have clarified this point further in the discussion:

*“Lower mean NI in AD suggests that, on average, removing a single node from the brain network will be less likely to suppress seizures in our simulations than in controls, and hence more distributed groups of nodes are likely to be responsible for driving seizures.”* (line 365-368)

*Why is the variability deviation of NI higher in controls than in AD? Given that the AD subgroup should be a more heterogeneous group, in particular when it comes to excitabilty and seizures, shouldn’t the variability in AD be higher? In the discussion the authors indicate that this could be driven by less spatial variability in AD than in controls, and this would seem a valid explanation when inspecting variability or STD across brain regions. However, figure4 A shows the average across all brain regions, so in principle region-to-region variability should not have an influence on the values. What could be the explanation?*

The results of figure 4 suggest that the value of NI in controls is less tightly constrained around its mean than in AD. This can arise due to differences in network topology as we previously demonstrated in [Lopes et al. (2017)]. For example, figure 5 of that paper shows that a small variance in NI can be seen in small world networks and a larger variance can be seen in scale free networks. That NI values would be more tightly distributed around the mean implies a lack of individual nodes that are most responsible for seizure generation, which fits into the picture of generalised seizures, as discussed.

*Paragraph starting 281. Authors are describing a hypothesis of the pathophysiology of AD, excitability and epileptogenesis. It is good to share such a hypothetical model in such a transparent way for the reader, but it should perhaps be clear at the start that this paragraph is stating an unproven hypothetical model.*

We have updated this paragraph to include the following statements:

“*While at present this is an untested hypothetic model, under this hypothesis we can interpret increased BNI in AD as follows…”* (line 322-323)

*“Future work should involve testing this hypothetical self-amplifying cascade within our model framework*.” (line 332-333)

*Additionally, the authors seem to be describing a model where excitability and seizure probability increase progressively across disease progression (moderate dementia subjects would have more probability of seizures than MCI subjects), but, is that really the case?*

Evidence in the literature suggests that this is the case. For an example, we refer the reviewer to Fig 1B of Beagle et al. (2017), which shows a plot of the seizure probability against years from disease onset, demonstrating a steady increase over disease progression. We have referred to this in the discussion:

*“It would be particularly interesting to track the progression of modelled seizure probability as brain networks evolve during disease progression. Indeed, the probability of seizures has been observed to increase steadily from disease onset [70].”* (lines 333-335)

*The authors seem to be implying that their AD subjects have no seizures and may develop seizures in the future, but they are “normal” at the time of EEG acquisition. However, various studies indicate that AD studies without known seizures may have substantial epileptiform activity, especially during night, but that one would not notice unless one does long full night EEG recordings or even better implanted electrodes in the medial temporal lobe to have enough sensitivity to inspect local medial temporal lobe activity. If the AD patients included in this study have no clinical history of seizures but have not undergone an in depth evaluation, it does not seem unlikely that some could have seizures / epileptiform activity that have not been detected.*

*References:
https://onlinelibrary.wiley.com/doi/abs/10.1002/ana.24794
https://n.neurology.org/content/95/16/e2259.abstract*

We thank the reviewer for bringing this interesting detail to our attention, and have added the following to highlight this point:

*“All participants had no clinical history of seizures, but no extensive electrophysiological workup was performed to definitely rule out subclinical epileptiform activity [49,50]”* (lines 93-95)

*Methods for PLV calculation. Line 129 states “ PLV values with zero phase-lag were rejected. Edges with a stronger indirect path were also rejected”. The phase lag will rarely be exactly zero so I imagine there are some thresholds to assess this. Could you share more details on this? Also on the direct and indirect paths are computed and compared.*

The threshold for phase lags corresponded to the phase difference corresponding to a single sample at the minimum bandpass frequency (in this case 4 Hz as the theta band ranges from 4-8 Hz). The Dijkstra algorithm was used to compute the length of the shortest path between any pair of nodes, and “indirect paths” corresponded to those with a shortest path which was not the single edge between the pair of nodes. We have updated the text to clarify this:

*“Zero-phase lag here corresponds to mean phase differences less than the phase difference equal to one sample at 4 Hz.”* (lines 134-135)

*“The Dijkstra algorithm was used to compute the length of the shortest path between all pairs of nodes, and edges with an indirect shortest path (i.e. the shortest path is not the single edge between the pair of nodes) were also rejected [27].”* (lines 135-138)

**Reviewer #2** *Major:
1. The authors use age and sex matched controls. The sex-matching, however, is not perfect (14 males in controls vs 8 males among case). Statistically, the sex difference was not significant. However, sex differences in connecivity have been reported across different imaging modalities - sometimes being introduced as artifacts from brain morphology. Thus, the sex-imbalance might influence the results here as well. (e.g., bias brain morphology on source localization or genuine sex differences in connectivity).
I suggest to conduct a supplementary analysis with a subsable with perfect sex matching to assess whether results remain significant (or if failing that, at least show the same dirction/pattern).*

The reviewer is correct that genders are not perfectly matched. We have repeated the analysis using the first 8 males from each group and the first 12 females, such that both cases and controls include equal numbers of males and females. Here, the ‘first’ X patients are the X patients with earliest date of data acquisition (i.e. equivalent to if we had stopped data acquisition after 8 males and 12 females for each group), chosen to avoid potential bias that could arise from manually selecting which participants to use and reject. In the original data, the effect sizes and p-values for the comparison of BNI scores were U=403, z=2.7710, p=0.0056, with a plot shown in Fig 2A in the manuscript demonstrating that BNI was larger in cases than controls on average. Below we have recreated this figure for perfectly gender-matched groups. The effect sizes and p-values were U=302, z=2.7456, p=0.0060, with trends in the same direction. Hence, the results demonstrated in this manuscript are not due to gender imbalance.

*2. In the MVPA approach, Logistic Regression is used. Was there any regularization used? There were 40 regions of interest (ROIs) and 49 samples, not making for an overly robust analysis, especially given that 5-fold CV was carried out, thus providing ~40 samples per training fold.*

No regularization was used for the MVPA approach. We highlight here that the aim of MVPA was not to develop a classifier for AD which is robust and generalizable to a new population of data. Instead, the aim of the analysis was to test whether the patterns of NI/nNI distributions significantly differed across groups by permuting the labels and recalculating the classification score (i.e. AUC) for the same dataset. Hence, we are less interested in the absolute value of AUC than how the AUC value compares against the permuted data. Each permutation uses the same cross-validation folds and re-fits a new logistic regression model to the permuted data, and hence the degree of overfitting is equal in the original and permuted data sets. Therefore any drop in classification score in the permuted data is likely due to the loss of some association between the NI/nNI distributions and the AD/control label. Hence, overfitting is not problematic for this analysis. We have highlighted this in the manuscript:

*“No regularization or feature selection was used to reduce overfitting, and hence MVPA classification rates may not be robust or generalizable to new populations of data. However, each permutation used the same cross-validation folds and hence the degree of overfitting is equal in the original and permuted data sets. Therefore any difference in AUC between permuted and empirical data suggests that there is an association between NI/nNI distribution and disease group in the empirical data which is not present after permutation.”* (line 233-239)

*Moreover, could the authors clarify the phrase “20 repetitions of this procedure were performed and the best performing repetition was used in subsequent analysis” . it is not clear what the subsequent analysis is (next part is the results section).*

Subsequent analysis referred only to the statistical testing via permutation analysis, and has been clarified in the manuscript:

*“20 repetitions of this procedure were performed and the average AUC was used in subsequent analysis (i.e. statistical testing via permutation analysis, described below).”* (line 227-229)

*Also selecting the best run out of 20 repetitions appears to be some sort of overfitting.*

We thank the reviewer for highlighting this mistake on our part. As can be seen in the mv_calculate_performance function of the MVPA-Lite toolbox and the sentence following the one highlighted by the reviewer in the manuscript, the value of AUC reported/used for permutation testing was *mean* and not *max* as this line of our manuscript suggests. We have corrected this line appropriately (see the response to the above comment for updates in the text).

*In addition, reporting AUC as mean and SE across CV folds is fine, but also across repetitions is leading to artificially small confidence intervals. Since SE depends on the sample size - and one can arguably run just more repetitions to get the SE as small as desired.*

To adjust for this, we have updated the manuscript to report standard deviation as opposed to standard error. The interpretation differs (i.e. it is a measure of variability as opposed to a measure of confidence in the estimate of the mean), but there is no longer a dependence on sample size. The methods and results have been updated to reflect this change.  *3. The authors state themselves that one “limitation of the modelling methodology is the use of a static functional network which is independent of cortical excitability.” In fact, this reviewer has the feeling that the analysis became detached from the original question about cortical excitability and seizures. This is overall a very, very evolved analysis and the only important parameter that differs between cases and controls is the adjacency matrix. This leads to the question of whether the results should be interpreted in terms of excitability and seizure potential.*

The reviewer is correct: the analysis that we have performed uses a model to quantify the excitability of a given network. Here we have quantified differences in overall excitability (BNI) and differences in the spatial distribution of mechanisms of excitability (NI) between AD and healthy controls. Using the model allows to quantify the excitability profile of the given network topology, so it is our intention to interpret the results this way. In order to clarify the point we intended to make, we have revised this passage as follows:

*“A limitation of the modelling methodology is the use of a static functional network which is independent of cortical excitability. This separation of local and network mechanisms has been shown to be informative in previous applications to epilepsy [27,39,40,43,87]. It lies between a standard functional connectivity analysis on the one hand, and the full inversion of a biophysical model on the other. The latter would simultaneously estimate intrinsic excitability of nodes and connectivity between them, thereby capturing the role of local dynamics in shaping large-scale functional connectivity [103]. However, it is a challenging inverse problem.*

*In addition, future work involving more biophysically realistic modelling could incorporate activity dependent degeneration [17], in which both the local dynamics and large-scale connectivity between populations are altered along the simulated disease progression. Our modelling also assumes homogeneity of local dynamics across regions of the brain, which is also likely to be of limited biophysical realism. Integration with other imaging modalities would allow for spatial heterogeneity in local excitability related to statistics such as amyloid/tau burden (PET scans) or neurodegeneration (cortical atrophy via structural MRI).”* (lines 461-477)

*Overall, many studies have demonstrated different functional connectivity profiles between AD and controls - predominantly around the default mode network - across a wide array of network derived properties. How can the authors ensure that the observed change of BNI is not simply related to one of the classic network properties?*

As stated above, the model allows to link network properties to seizure generation. Indeed, in the wider context of epilepsy we have studied the relationship between BNI and NI and different measures of network topology [Lopes et al. (2017); Laiou et al. (2019)]. In the context of AD, we performed a full graph theoretical analysis of these networks in Tait et al. (2019), demonstrating some differences. However, graph theoretical metrics can only quantify differences between network structures, but a modelling framework such as the one presented here allows to test a specific hypothesis, e.g. in the current study, we use simulations to relate how these alterations in functional connectivity may lead to increased seizure propensity in AD and quantify the importance of different nodes in the network for seizure generation. These specific hypotheses cannot be tested by graph theoretical functional connectivity analysis alone.

*To be more convincing in this regard, the work would need some verifiable predictions (e.g., “people with highest BNI did actually evolve seizures”), or at least some form of specificity analysis e.g., compare the findings here to another analysis between controls and patients with another brain disorder (with aftropy) but that does not exhibit seizures. Is the BNI here as well different between cases and controls? Other options could be, e.g., among controls to compare men and women or test the association between BNI and age - in these analysis one would not - per se - expect differences in BNI.*

We agree, and have highlighted this point in the “Limitations and Future Work” section in much detail. We highlight e.g. that these results can only be interpreted with this data as a group level effect and not as individual level predictors. In future work we would aim to study the above questions in relevant AD cohorts, though we do not currently have such data. We note however that the framework we use (BNI and NI) has been validated in the context of epilepsy by distinguishing people with epilepsy vs controls [Petkov et al. (2014); Lopes et al. (2021)], differentiating generalised from focal epilepsy [Lopes et al., (2019)], predicting changes in BNI due to surgical removal of network nodes [Goodfellow et al. (2016); Laiou et al. (2019)], and to lateralise the epileptogenic zone from scalp EEG [Lopes et al. (2020)].

*Minor:
1. In formula (2) the index appears to be incorrect: \theta_i should be \theta_j .*

We thank the reviewer for highlighting this typo, which has now been corrected in equation 2.

**Reviewer #3**

*This a very interesting study, focused on modeling/understanding the increased tendency of the AD brain to develop seizures. The authors used functional brain networks estimated from EEG data and generative computational modeling to simulate brain activity, where a given parameter allows to explore how the healthy control and AD brains will behave in terms of seizure generation. The study, and its findings, are of interest for the research community. I have, however, a few major comments that should be clarified before considering the manuscript for publication:

1) The procedure to construct individual functional brain networks from EEG data involve multiple empirical steps. For example, after source reconstruction, (i) filtering the signal to the theta band, (ii) removing connections not reaching the 95% significance, (iii) removing connections with strong indirect paths, etc. Each of these steps, can imply or cause obtaining different networks for NC and AD subjects (differences in EEG-frequency have been reported, strength of connections have also been documented, indirect effects may appear more often in one population than the other, etc). If the networks used as input on the phenomenological model are very different, it would not be surprising at all the obtained differences in seizure tendencies for NC and AD groups, which would appear just as a direct result of network reconstruction procedures (i.e. may result from artificial effects). The authors need to explore/clarify the impact of their network-reconstruction steps on the global and regional ictogenicity analyses.*

The reviewer is correct that changing the network pipeline can affect the ictogenicity outcome, and it has been studied in detail how different network structures can lead to different BNI outcomes [Lopes et al. (2017)]. In this study, we chose *a priori* a single pipeline that was appropriate for the novel scientific question at hand (ictogenicity in AD). Justification for the functional connectivity pipeline was discussed in great detail in the manuscript in the “Methodology” section of the discussion, but here we highlight that the methods used for construction of functional connectivity were taken directly from a previous study [Tait et al. (2019)] which did not consider seizure propensity or the theta model and hence the pipeline was not specifically cherry picked to obtain the optimum ictogenicity results. Tait et al. (2019) showed using graph theoretical metrics that using this methodology there were differences in functional connectivity between control and AD participants. Hence, the network methodology was chosen as it is known to differentiate AD from control. A similar pipeline was additionally used in a previous source-space study considering localization of seizure onset zone in people with epilepsy [Lopes et al. (2020)], demonstrating that such methods are useful for the model-based ictogenicity analysis used here.

We have now updated the Methodology section to highlight that BNI/NI scores may differ if different pipelines were used:

“*There are several methodological considerations to this study, as functional network structure is likely to be dependent on analysis pipeline and influences the BNI/NI results [40]. Here, we chose a priori a single pipeline that was appropriate to for the scientific question at hand. The methods used for construction of functional connectivity were derived from a previous study [33] which showed differences in functional connectivity between controls and AD using graph theoretical metrics. A similar pipeline was additionally used in a previous source-space study for BNI analyses demonstrating usefulness for the model-based ictogeneicity analysis [41].”* (lines 390-397)

*In addition, if the NC and AD networks would be matched in terms of number of connections (and their location), would the observed results still valid?*

Different individuals have different networks, and these individual networks are directly comparable as long as the same methodology is used to construct each for each participant. Indeed, in our study the networks are statistically different between AD and controls even though identical steps were taken to derive the networks [Tait et al. (2019)]. Since the only parameters of the model that change on an individual basis are the networks, these differences in functional connectivity directly underpin our results, i.e. in our model, differences in functional connectivity, together with a hypothesised increase in cortical excitability (which is the same across all participants), revealed that the AD group had a higher likelihood to develop seizures than the control group.

*2) Since the Introduction, the authors made an emphasis on the link between amyloid and tau deposition with the increased seizure tendency in AD. First, the are many other well-documented biological alterations that can be causally related to such effects. For example, vascular dysregulation, metabolic alterations and increased inflammation, resulting in neuronal activity dysregulation. The authors should also comment on those potential effects, as a way to make their study less biased towards a unique pair of biological factors (mostly towards amyloid). Avoiding an amyloid-centered and -limited view will significantly increase the value of the study.*

We thank the reviewer for this useful comment. We have updated the relevant sections of text to address this comment:

*“Additional key factors which may influence seizure likelihood in AD are vascular dysregulation, metabolic alterations and increased inflammation, resulting in neuronal activity dysregulation [24, 25]”* (lines 29-31)

*“Future work should involve integration of multi-modal neuroimaging data, including functional data (EEG/MEG/fMRI), structural MRI, and PET, to quantify the relationship between spatial patterns of NI and Alzheimer’s pathologies, cortical atrophy, vascular dysregulation, metabolic alterations, inflammation, etc. on an individual level”* (lines 456-460)

*“Integration with other imaging modalities such as PET/MRI would allow for spatial heterogeneity in local excitability related to statistics such as amyloid/tau burden, vascular/metabolic dysfunction, inflammation, or neurodegeneration.”* (lines 474-477)

*3) In relation with the previous comment, the link between increased regional ictogenicity in AD and amyloid deposition seems forced. The authors are finding a few areas that, yes, are among those usually identified with high amyloid burden. But, are the whole brain regional patterns similar? Can this be statistically tested?*

It is not possible, for this data set, to test the statistical associations between amyloid burden and regional ictogenicity as no amyloid PET scans were available for this cohort. This was previously highlighted and discussed in the “Limitations and future work” section of the discussion, where the relevant paragraph reads:

*“In this work, we identified spatial distributions of regions with high seizure propensity in AD, and observed a resemblance to stereotypical patterns of Abeta pathology in AD patients. A limitation to further exploring this observation and quantifying statistical effects if the absence of amyloid PET scans in our cohort. Future work should involve integration of multi-modal neuroimaging data…”* (lines 453-456)

To make this point more explicit, we have additionally updated the results/discussion to read:

*“Interestingly, as a group-level observation we note that these regions seem to be consistent with those with the largest and earliest staged Abeta burdens in AD [44, 45], but this was not tested statistically on the individual-level as no amyloid PET scans were available for our participants”* (lines 285-288)

*“These associations support a group-level relationship between seizure propensity and regions stereotypically affected by AD at the earliest stages, which should be tested on an individual-level using multimodal imaging in future work”* (lines 355-357)

*Amyloid deposition in the brain is very unspecific and widespread, almost all brain regions get amyloid at some point.*

The reviewer is correct that at later stages of the disease, amyloid burden does become very widespread. However, stereotypical patterns at early stages are described in the post-mortem amyloid staging of Braak and Braak (1991) and the *in vivo* staging of Grothe et al. (2017). These papers are cited throughout at the relevant locations in the text.

*This is different for tau, structural atrophy, and, as said, many other biological factors involved in AD pathomechanisms. In addition to comparing with whole brain regional amyloid patterns, could the authors compare with at least two other factors altered in AD?*

We highlight that this correspondence with stereotypical patterns of amyloid burden was a group-level observation and no statistical testing was performed. In the discussion of the manuscript, we compared against tau burden in the same regard:

*“In our model, therefore, the ROIs which have the potential to most strongly drive seizures are those stereotypically found to have the earliest and strongest Abeta burden in AD. These same regions are also those affected by tau burden at middling stages (Braak stages III-IV) of the disease [44]”.* (lines 346-349)

We have added a comparison with cortical atrophy immediately following these sentences:

*“Furthermore, Frisoni et al. (2009) [71] developed an in vivo staging of cortical atrophy in AD, and in the earliest stages reported 20-30% cortical grey matter loss in the medial temporal, posterior cingulate, and orbitofrontal cortices, which similarly align to the regions with largest NI in our model. However, a 15-20% loss of grey matter in the temporoparietal region was additionally reported which does not correspond to our results.”* (lines 350-354)

**References**

Beagle et al. (2017) J Alzheimers Dis 60(1):211-223, doi: [10.3233/JAD-170031](https://doi.org/10.3233/JAD-170031)

Braak and Braak (1991) Acta Neuropathol 82:239-259, doi: [10.1007/BF00308809](https://doi.org/10.1007/BF00308809)

Goodfellow et al. (2016) Sci Rep 6:29215, doi: [10.1038/srep29215](https://doi.org/10.1038/srep29215)

Grothe et al. (2017) Neurology 89(20):2031-2038, doi: [10.1212/WNL.000000000004643](https://doi.org/10.1212/WNL.000000000004643)

Laiou et al. (2019) Front Neurol 10:1045, doi: [10.3389/fneur.2019.01045](https://doi.org/10.3389/fneur.2019.01045)

Lopes et al. (2017) PLoS Comput Biol 13(8):e1005637, doi: [10.1371/journal.pcbi.1005637](https://doi.org/10.1371/journal.pcbi.1005637)

Lopes et al. (2020) Clin Neurophysiol 131(1):225-234, doi: [10.1016/j.clinph.2019.10.027](https://doi.org/10.1016/j.clinph.2019.10.027)

Lopes et al. (2021) Clin Neurophysiol 132(4):922-927, doi: [10.1016/j.clinph.2020.12.021](https://doi.org/10.1016/j.clinph.2020.12.021)

Petkov et al. (2014) Front Neurol 5:261, doi: [10.3389/fneur.2014.00261](https://doi.org/10.3389/fneur.2014.00261)

Tait et al. (2019) Clin Neurophysiol 130(9):1581-1595, doi: [10.1016/j.clinph.2019.05.027](https://doi.org/10.1016/j.clinph.2019.05.027)
