# Supplementary material for: A large-scale brain network mechanism for increased seizure propensity in Alzheimer’s disease
Source: PLoS Comput Biol. 2021 Aug 11;17(8):e1009252. doi: 10.1371/journal.pcbi.1009252 (PMC8382184; doi:10.1371/journal.pcbi.1009252)
Supplement: Attachment — Submitted filename: AD-BNI_Reviewer_response_2_ML.docx [file pcbi.1009252.s006.docx]

**Reviewer 1**

1. We thank the reviewer for highlighting some important points. From our perspective the Figure suggested by the reviewer also does not answer the question of seizure likelihood through disease progression, since the figure they present shows raw numbers of patients with new-onset seizures. Naturally this will show smaller numbers as years relative to onset increase for a range of reasons, including the fact that there are likely to be fewer participants who survive as time progresses and fewer participants who have not already developed seizures. A middle ground between the figure we suggested (which shows cumulative probability, i.e. normalized to total count) and the figure the reviewer suggested would be required to answer the question sufficiently, as this would show the probability of developing over time (as opposed to the cumulative probability). Unfortunately, as far as we are aware no such evidence is presently available.

   We have therefore removed the claim of a relationship between seizure likelihood and disease staging and instead clarified that this relationship is unclear. We additionally highlighted this as an interesting avenue for future work. This sentence now reads: *“It would be particularly interesting to longitudinally track the evolution of BNI throughout cognitive decline, as it is currently unclear whether seizure likelihood evolves with disease progression.”* (line 335-336)

   We additionally wish to highlight that whether the point under scrutiny is true or false (or unknown) is not important for our model assumptions. In our model, we only assume that cortical excitability increases in AD and not that this increase is related to disease progression. For example, in our caption to Figure 2 we state *“The black arrow shows a hypothetical ‘current’ baseline, in which no participants have seizures. The yellow arrow shows* ***that if cortical excitability increases****, AD participants are more likely to develop seizures than controls”* (emphasis added). We do not make assumptions about when in disease progression increases in cortical excitability occur, just that at present none of our participants have seizures and, in our simulations, if cortical excitability is increased the AD participants are more likely to develop seizures. Similar statements/arguments are made throughout the results (e.g. lines 251-258) and discussion. Statistically, on a group level, people with AD are more likely to develop seizures than controls, and our model suggests a large-scale network-level mechanism for this observation. The evidence supporting increased cortical excitability in AD is reviewed in great detail in the introduction and discussion.
2. We have clarified this wording further to reduce any potential confusion. The sentence now reads “Zero-phase lag here corresponds to a mean phase difference smaller than the phase resolution at 4 Hz, given the sampling rate, i.e. if the mean phase difference was less than ($2\pi\times4)/f_{sample}=0.008\pi$ radians, the edge was set to zero.” (lines 134-136)

**Reviewer 2**

We have changed line 99 to read “sex” instead of “gender” as suggested by the reviewer.
